# Heat Stress Drives Rapid Viral and Antiviral Innate Immunity Activation in Hexacorallia

**DOI:** 10.1111/mec.70098

**Published:** 2025-09-07

**Authors:** Ton Sharoni, Adrian Jaimes‐Becerra, Magda Lewandowska, Reuven Aharoni, Christian R. Voolstra, Maoz Fine, Yehu Moran

**Affiliations:** ^1^ Department of Ecology, Evolution and Behavior, Alexander Silberman Institute of Life Sciences Faculty of Science, The Hebrew University of Jerusalem Jerusalem Israel; ^2^ Department of Biology University of Konstanz Konstanz Germany; ^3^ The Interuniversity Institute for Marine Sciences Eilat Israel

**Keywords:** climate change, cnidarians, immunity, stress, viruses

## Abstract

The class Hexacorallia, encompassing stony corals and sea anemones, plays a critical role in marine ecosystems. Coral bleaching, the disruption of the symbiosis between stony corals and zooxanthellate algae, is driven by seawater warming and further exacerbated by pathogenic microbes. However, how pathogens, especially viruses, contribute to accelerated bleaching remains poorly understood. Here the model sea anemone *Nematostella vectensis* is used to explore these dynamics by creating a transgenic line with a reporter gene regulated by sequences from two RIG‐I‐like receptor genes involved in antiviral responses. Under heat stress, the reporter genes showed significant upregulation. Further, transcriptomes from *N. vectensis* , *Exaiptasia diaphana* and the stony coral *Stylophora pistillata* were analysed to reveal stress‐induced activation of a set of *bona fide* immune‐related genes conserved between the three species. Population‐specific differences in stress‐induced transcriptional responses of immune‐related genes were evident in both *Nematostella* and *Stylophora*, depending on geographic origin. In *Exaiptasia*, the presence of zooxanthellae also influenced stress‐induced immune gene expression. To test whether the viruses themselves contribute to this immune response under stress, we subjected *N. vectensis* to heat stress and measured the transcription dynamics of resident viruses as well as selected antiviral genes. While the antiviral genes responded within hours of heat stress, viral gene expression was already upregulated within 30 min, suggesting that their increase might be contributing to the elevated immune response under stress, and consequentially, the further demise of organismal homeostasis. These findings highlight the interplay between environmental stress, viruses, immune responses and symbiotic states in Hexacorallia.

## 1 Introduction

Coral reefs are a habitat supporting at least 25% of all marine species (Fisher et al. 2015; Hoegh‐Guldberg et al. 2019). They are formed by members of the order Scleractinia, also known as stony corals, which are dependent on close symbiosis with zooxanthellae, dinoflagellates in the family Symbiodiniaceae that reside within their gastrodermis (Fransolet et al. 2012; LaJeunesse et al. 2018; Jacobovitz et al. 2023). Coral bleaching, driven by symbiont loss, has escalated alarmingly in recent decades, with global events increasingly frequent and severe (Sully et al. 2019; van Woesik et al. 2022), leading to extensive habitat loss (Reimer et al. 2024).

The exact causes underlying coral bleaching are still under debate and this seems to be a complex phenomenon that may be attributed to numerous different factors acting in parallel (Rädecker et al. 2021; Helgoe et al. 2024; Schlotheuber et al. 2024). Yet, there is a growing consensus that the rise of water temperature is a major cause of this via the induction of coral stress (Cziesielski et al. 2019) that leads to the collapse of nutrient cycling between the algal symbiont and coral host and the buildup of reactive oxygen species (ROS) triggering oxidative damage (Downs et al. 2002; Rädecker et al. 2021, 2023). Another potential cause frequently suggested as contributing to bleaching is pathogenesis, mostly by bacteria (Rosenberg et al. 2007). How exactly the stress and/or pathogenesis results in the loss of zooxanthellae is still being investigated and one of the major approaches in the field has been differential gene expression analysis, comparing healthy and bleached corals (DeSalvo et al. 2008; Seneca et al. 2010; Bellantuono et al. 2012; Pinzón et al. 2015; Seneca and Palumbi 2015). Multiple studies in stressed and bleached corals observed strong upregulation of genes that are homologous to known immune‐related genes from vertebrates (DeSalvo et al. 2008; Kenkel et al. 2014; Rosic et al. 2014; Pinzón et al. 2015; Wang et al. 2024). Thus, current evidence suggests a potential link in corals between stress, pathogenesis and immunity. Two non‐mutually exclusive scenarios may explain the link between stress and immune response: in the first, heat stress induces pathogenesis that in turn induces the immune response. Alternatively, in the other scenario, the immune system of corals evolved to be upregulated upon heat stress as this would be advantageous against pathogens that tend to be more active under heat stress.

Although work on coral in their natural environment is invaluable to study bleaching, a detailed cell biological understanding cannot be achieved without manipulative experiments in a controlled lab environment, which prompts the use of model organisms (Weis et al. 2008). Scleractinia are part of the cnidarian class Hexacorallia, which also includes sea anemones (Ruppert et al. 2004). Sea anemones offer practical advantages as laboratory models due to their ease of culture and controlled reproduction. Some were developed into lab model organisms for answering questions in evolutionary developmental biology (‘evo‐devo’) and ecology (Darling et al. 2005; Baumgarten et al. 2015; Rentzsch et al. 2017; Al‐Shaer et al. 2021; Röttinger 2021; Jacobovitz et al. 2023; Roberty et al. 2024). The two arguably most developed sea anemone model species are *Nematostella vectensis* and *Exaiptasia diaphana* (previously called *Aiptasia pallida* , frequently referred to as Aiptasia). While both *N. vectensis* and *E. diaphana* are separated by roughly 500 million years from stony corals (Erwin et al. 2011; Shinzato et al. 2011), the latter harbours zooxanthellae in their gastrodermis just like corals, making it a useful model for studying hexacorallian–dinoflagellate symbiosis (Jacobovitz et al. 2023; Roberty et al. 2024).

*N. vectensis* lives in distinct populations spread across isolated brackish lagoons on the Atlantic coast of North America from Canada to southern USA (Reitzel et al. 2013). There is little gene flow between these populations, and yet we have shown that despite highly similar genomic sequences (Smith et al. 2023), they evolved different transcriptional responses to environmental stress (Sachkova et al. 2020). Similarly, we have shown that populations of the stony coral *Stylophora pistillata* , originating either from the Gulf of Aqaba (GoA) at the northern point of the Red Sea or from the central Red Sea (CRS), exhibit different transcriptional responses to heat stress (Voolstra et al. 2021), but it is unclear whether they form distinct genetic populations (Buitrago‐López et al. 2023). Similar trends were also reported between species (Voolstra et al. 2023). Interestingly, allelic variability was reported for the key immune‐ and stress‐related transcription factor NF‐ κB between populations of *N. vectensis* (Sullivan et al. 2009). Thus, interspecific and intraspecific genetic variation in Hexacorallia may drive different responses to environmental cues, possibly reflecting local adaptation. Yet, whether this variation has an effect on immunity is currently unknown.

A recent study showed that under heat stress conditions phagocytic activity is increased in hexacorallians (Eliachar et al. 2022). Moreover, key players in the immune response are downregulated in hexacorallians during symbiosis with zooxanthellae (Mansfield and Gilmore 2019). While much attention was given to the effect of bacteria on the hexacorallian immune response (Bourne et al. 2009; Mao‐Jones et al. 2010; Munn 2015), far less attention was provided for viral agents. Yet, there are indications that upon heat stress, herpes‐like viruses present in the coral are activated and the amount of the virus increases (Vega Thurber et al. 2008). Moreover, there are indications that viruses that are pathogenic to the zooxanthellae become more productive under higher water temperatures (Levin et al. 2016; Howe‐Kerr et al. 2023). Thus, viruses and the immune response that defends against them may play an underappreciated role in shaping symbiosis stability in Hexacorallia.

We have started revealing the antiviral innate immune system of *N. vectensis* by characterising the transcriptomic response of this species to double‐stranded RNA (dsRNA), a pathogen‐associated molecular pattern (PAMP) typifying most viruses (Lewandowska et al. 2021). We have shown that this viral hallmark is detected by the cytosolic receptor RIG‐I‐Like Receptor b (RLRb), a homologue of the mammalian RIG‐I and MDA5 receptors that is conserved in all hexacorallians (Lewandowska et al. 2021; Iwama and Moran 2023). The transcriptional response we reported is diverse and includes upregulation of homologues of genes of the vertebrate interferon response as well as those known to participate in antiviral RNA interference (RNAi) in protostomes, such as insects and nematodes. Similarly, a wide transcriptional response was detected in *N. vectensis* upon immune system activation by 2′3′‐cGAMP. This cyclic dinucleotide is known to serve as a signalling molecule in the antiviral immune system of vertebrates, and this study highlighted that concomitantly homologues of antibacterial genes are upregulated by this challenge in this hexacorallian species (Margolis et al. 2021). Currently, very little is known about how the immune systems of other hexacorallians react to viral PAMPs.

In this work, we have set to explore how stress is linked to antiviral innate immunity by analysing existing transcriptomic resources from three major hexacorallian models, the sea anemones *E. diaphana* and *N. vectensis* and the stony coral *S. pistillata* . Additionally, we utilised transgenesis tools established for *N. vectensis* (Renfer et al. 2010; Renfer and Technau 2017) as well as the knowledge about its core virome (Lewandowska et al. 2020) to further explore this intriguing link.

## 2 Materials and Methods

### 2.1 Sea Anemone Culture

*N. vectensis* early life stages (embryos, larvae and primary polyps) were grown in the dark at 22°C and in a salinity of 16‰ artificial seawater, whereas juveniles were grown at 18°C. From 2 weeks after fertilisation, the polyps were fed with *Artemia salina* nauplii three times a week. Induction of gamete spawning was performed as previously described (Genikhovich and Technau 2009). For performing microinjection, the fertilised eggs were separated from the egg package by incubation in 3% l‐cysteine (Merck Millipore, Burlington, MA). For all other usage, the fertilised eggs were left for 3 days at 22°C until the developed planulae were spontaneously released from the gelatinous egg sack. All *N. vectensis* individuals used in this study were of the common lab strain originating from Rhode River, Maryland, USA (Hand and Uhlinger 1992).

### 2.2 Cloning and Transgenesis

The promoter regions of *NveRLRa* (scaffold_15:1,087,962–1,091,460; coordinates are taken from (Putnam et al. 2007)) and *NveRLRb* (scaffold_40:697,555–699,446) genes were amplified by employing the Advantage 2 polymerase (Takara‐Bio, Kusatsu, Japan) from a genomic DNA template. The backbone vector for these transgenesis experiments was a pCR2.1 vector (Thermo Fisher Scientific, Waltham, MA, USA) modified by the Technau lab (University of Vienna, Austria) holding a cis‐regulatory element, driving a gene encoding the mCherry fluorescent reporter protein (Shaner et al. 2004) and a SV40 viral sequence for polyadenylation signalling (Renfer et al. 2010; Renfer and Technau 2017). The PCR fragments were amplified so they would carry *PacI* and *AscI* cloning sites which are included in the vector. After overnight digestion with the two restriction enzymes (New England Biolabs, Ipswich, MA, USA) ligation of the vector and insert was performed with T4 DNA Ligase (New England Biolabs) and the ligation products were chemically transformed to NEB5α Competent Cells (New England Biolabs) according to the manufacturer's protocol. Purification of plasmids was done by HiSpeed Plasmid Midi Kit (Qiagen N.V., Venlo, Netherlands) and validated by Sanger sequencing (performed at the Genomic Technologies Center of the Hebrew University of Jerusalem). The plasmid of each RLR gene was injected into *N. vectensis* zygotes along with the meganuclease I‐*Sce*I (New England Biolabs) to enable genomic integration as previously described (Renfer et al. 2010; Renfer and Technau 2017). Transgenic animals were visualised under an SMZ18 stereomicroscope equipped with a DS‐Qi2 camera (Nikon, Tokyo, Japan) and positive animals were selected and reared to the adult stage. To sort the positive animals, we incubated planulae at 37°C for 24 h to stimulate the gene expression of the NveRLRa and NveRLRb resulting in the expression of mCherry (see Section 3). In each generation, we crossed transgenic animals with wild type animals and tested the offspring to find positive animals. Then we crossed the positive siblings until we got adult positive homozygous animals (F3). Sequences of all used primers are provided in Table S1.

### 2.3 Injection of Viral Mimics to Transgenic Animals

To initiate the response of the antiviral immune system in *N. vectensis* , we used dsRNA as a mimic of viral RNA. We used 6.25 ng/mL of high molecular weight (HMW) Polyinosinic:polycytidylic acid (poly(I:C)), a commercially available synthetic dsRNA, in 0.9% NaCl (Invivogen, San Diego, CA, USA) with an average size of 1.5–8 kb, and 0.9% NaCl as a control. The concentration was selected after a series of injections which we found to be the most efficient to test the antiviral immune response. Higher concentrations resulted in high mortality and aberrant zygote morphology after 24 h. In each experiment, 150–200 zygotes were injected per group and kept at 22°C. Transgenic animals were visualised under an SMZ18 stereomicroscope equipped with a DS‐Qi2 camera (Nikon).

### 2.4 Dynamic Heat Stress Assay

Wild type adult *N. vectensis* polyps were incubated for different time periods at 37°C (basal condition 0, 0.5, 1, 3, 6, 24, 48 h). For each time point, four biological replicates were prepared. Per each biological replicate, 4–6 animals were flash‐frozen in liquid nitrogen, ground and stored at −80°C until further processed. We conducted the RNA extraction using Trizol (Thermo Fisher Scientific) according to the manufacturer's protocol. cDNA was constructed using the iScript cDNA Synthesis Kit (Bio‐Rad, Hercules, CA, USA) according to the manufacturer's protocol. Real‐time PCR was prepared with Fast SYBR Green Master Mix (Thermo Fisher Scientific) on the QuantStudio 3 Real‐Time PCR System (Thermo Fisher Scientific). Six genes and six viruses (the sequences of the viruses are located in Data S1) were chosen for detecting the change during the heat stress assay. The genes are related to different pathways of the immune system, such as Interferon and RNA interference (Lewandowska et al. 2021). The six virus sequences chosen from the core virome assembly (Lewandowska et al. 2020), by their abundance in the viral load, altogether comprise ~80% of the total viral load. The relative expression was calculated by the relative standard curve method, and the relative comparison was to the basal condition. We converted the Ct value to fold change (2^x^) and compared the ratio between the increase in the viral load or the gene expression level to the basal condition state. Sequences of all primers and statistical tests are provided in Tables S2 and S3.

### 2.5 Differential Gene Expression Analyses

To assess the impact of heat stress on immune system activation, we reanalysed data from four previously published studies focused on hexacorallian species within the phylum Cnidaria. These studies share a common emphasis on examining transcriptomic responses to natural environmental stressors, particularly heat stress. Two of the studies involve populations of the sea anemones, *N. vectensis* (Sachkova et al. 2020) and *E. diaphana* (Cleves et al. 2020). The remaining two studies examine the stony coral species *S. pistillata* (Savary et al. 2021; Voolstra et al. 2021). All four studies follow a similar experimental framework. Individuals were exposed over short time frames to a defined control or baseline temperature and to one or more elevated temperatures (typically ranging from 2°C to 16°C above the control) designated as heat stress conditions. Differential gene expression analyses were then performed using RNA‐seq to compare the control and stress groups. Sachkova et al. (2020) exposed *Nematostella vectensis* from North Carolina and Massachusetts to 36°C for 24 h, using 20°C as the control, and identified transcriptional changes, including shifts in venom gene expression. Cleves et al. (2020) investigated gene expression dynamics in *E. diaphana* under 34°C heat stress, in both symbiotic and aposymbiotic states, relative to a 27°C control, with the aim of identifying genes associated with bleaching. Savary et al. (2021) exposed *S. pistillata* from the Gulf of Aqaba to short‐ and long‐term heat stress up to 34.5°C, with a control of 27°C, and analysed changes in both gene expression and microbial communities. Voolstra et al. (2021) subjected *S. pistillata* populations from the Gulf of Aqaba and the central Red Sea to acute heat stress (30°C control vs. 33°C, 36°C and 39°C) and compared transcriptomic responses between populations within each region following an 18‐h short‐term stress assay.

### 2.6 Orthology Analysis to Identify Immune‐Related Genes

We curated 56 *bona fide* immune‐related genes significantly upregulated following poly(I:C)‐induced antiviral challenge in *N. vectensis* . polyI:C is a mimic of long viral dsRNA and a primary ligand for the vertebrate RLR melanoma differentiation‐associated protein 5 (MDA5) (Lewandowska et al. 2021). Next, we conducted gene ontology analysis and revealed their relation to immune‐related processes. These genes showed homology to different key components of various immune pathways in *Homo sapiens* , including RNA interference, the Interferon pathway, the cGAS–STING pathway, among others. In the five studies we used for our analyses, these genes were used as markers to identify the triggered transcriptomic immune response when the individuals were subjected to heat stress. Since these genes were originally identified in *N. vectensis* , an orthology analysis was conducted to identify their corresponding orthologs in the genomes of *E. diaphana* and *S. pistillata* . OrthoFinder v. 2.4.0 (Emms and Kelly 2019) which combines BLAST and phylogenetics for high accuracy was used to identify orthogroups and orthologs between the *E. diaphana* and *S. pistillata* genomes. Additionally, the identified orthologs were analysed for their domain structures using InterProScan (https://pubmed.ncbi.nlm.nih.gov/24451626/) to validate and confirm the accuracy of the orthology inference. Only sequences that exhibited similar domain architectures across the genomes of the three species were accepted as orthologous for the corresponding immune‐related genes. The list of immune‐related genes for *N. vectensis* and their respective orthologs in *E. diaphana* and *S. pistillata* are provided in Table S4, with the corresponding sequences available in Table S5.

### 2.7 RNA‐Seq Data Processing

Raw reads were downloaded from four studies (Table S15) and were analysed using an identical strategy to identify immune‐related genes (from the list of 56 bona fide genes) that are upregulated in animals subjected to heat stress compared to control animals. The quality of reads was checked with FastQC (Andrews 2010) before and after read trimming with Trimmomatic version 0.36 (Bolger et al. 2014). Reads were aligned to the *N. vectensis* (Putnam et al. 2007), *E. diaphana* (Baumgarten et al. 2015) and *S. pistillata* (Voolstra et al. 2017) genomes using STAR under default alignment parameters (Dobin et al. 2013). Read counts matrices were generated using feature counts (Liao et al. 2014) using default parameters. Differentially expressed genes (DEGs) were determined based on Deseq2 (Love et al. 2014) and EdgeR (Robinson et al. 2010) packages. Genes with log_2_ fold changes ≥ 1 and adjusted *p* value ≤ 0.05 were considered to have a significant differential expression. To ensure high confidence in the results, we focused on the intersection of genes identified as significantly differentially expressed by both methods.

Volcano plots were generated based on DESeq2 results to visualise the results of differential expression in *N. vectensis* , while heatmaps were used to display the log2 fold change (LFC) in immune‐related gene expression across baseline and stress temperatures for each of the studies analysed. If any of the 56 evaluated immune‐related genes did not meet the defined thresholds, it was assigned a value of 0 and represented as dark cyan in the heatmap. The analysis workflow, implemented as a R script for differential expression analysis is available as File S2.

## 3 Results

### 3.1 *RLRa* and *RLRb* Promoters Are Sensitive to a Viral PAMP and Stress

**FIGURE 1:**
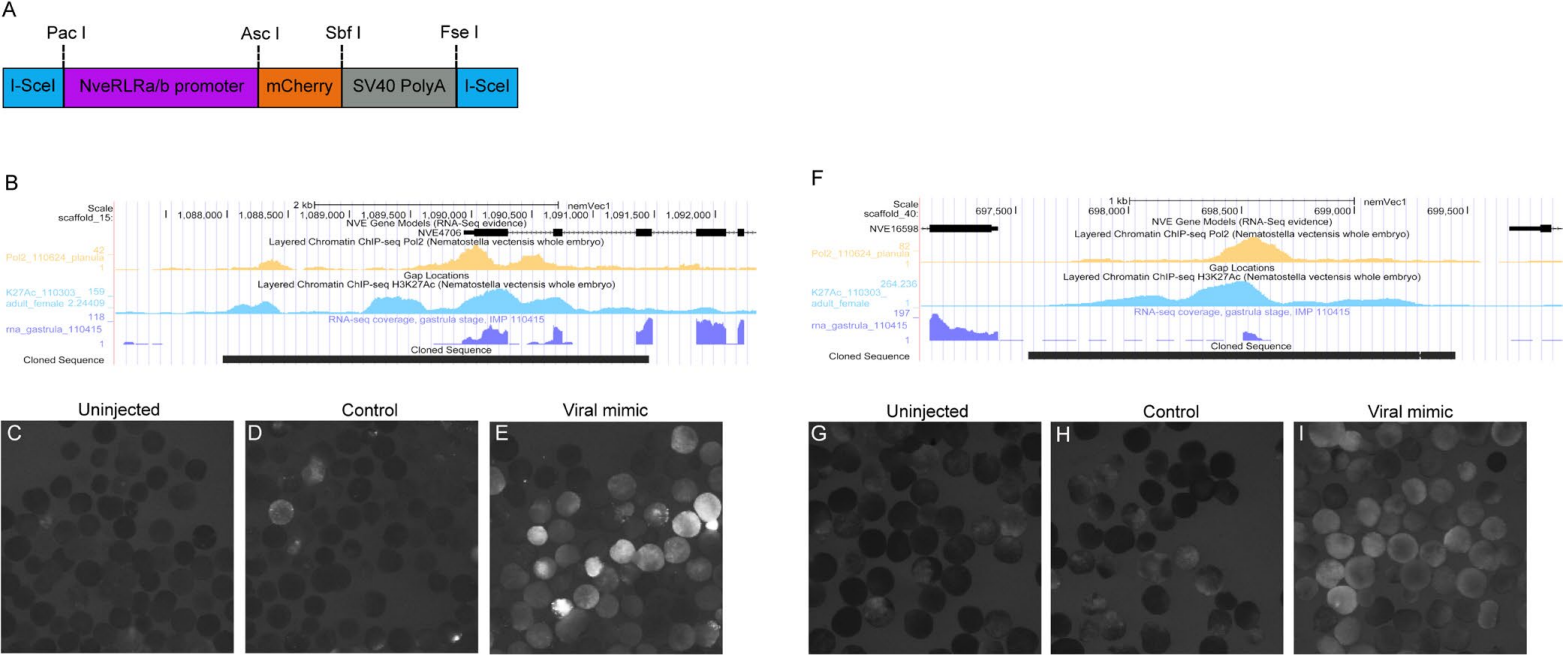
Generation of the transgenic *RLRa* and *RLRb* reporter lines and their response to dsRNA microinjection. (A) The design of the microinjected cassette used to generate the transgenic *N. vectensis* lines. (B) Features of the genomic segment containing the *RLRa* regulatory region. (C–E) The expression of mCherry in the *RLRa* transgenic reporter line. (C) Uninjected animals, (D) NaCl injected animals, (E) poly(I:C) injected animals. (F) Features of the genomic segment containing the *RLRb* regulatory region. (G–I) The expression of mCherry in the *RLRb* transgenic reporter line. (G) Uninjected animals, (H) NaCl injected animals, (I) poly(I:C) injected animals. The *RLRa* transgenic reporter line was documented 19 h after fertilisation, and the *RLRb* transgenic reporter line was documented 24 h after fertilisation.

**FIGURE 2:**
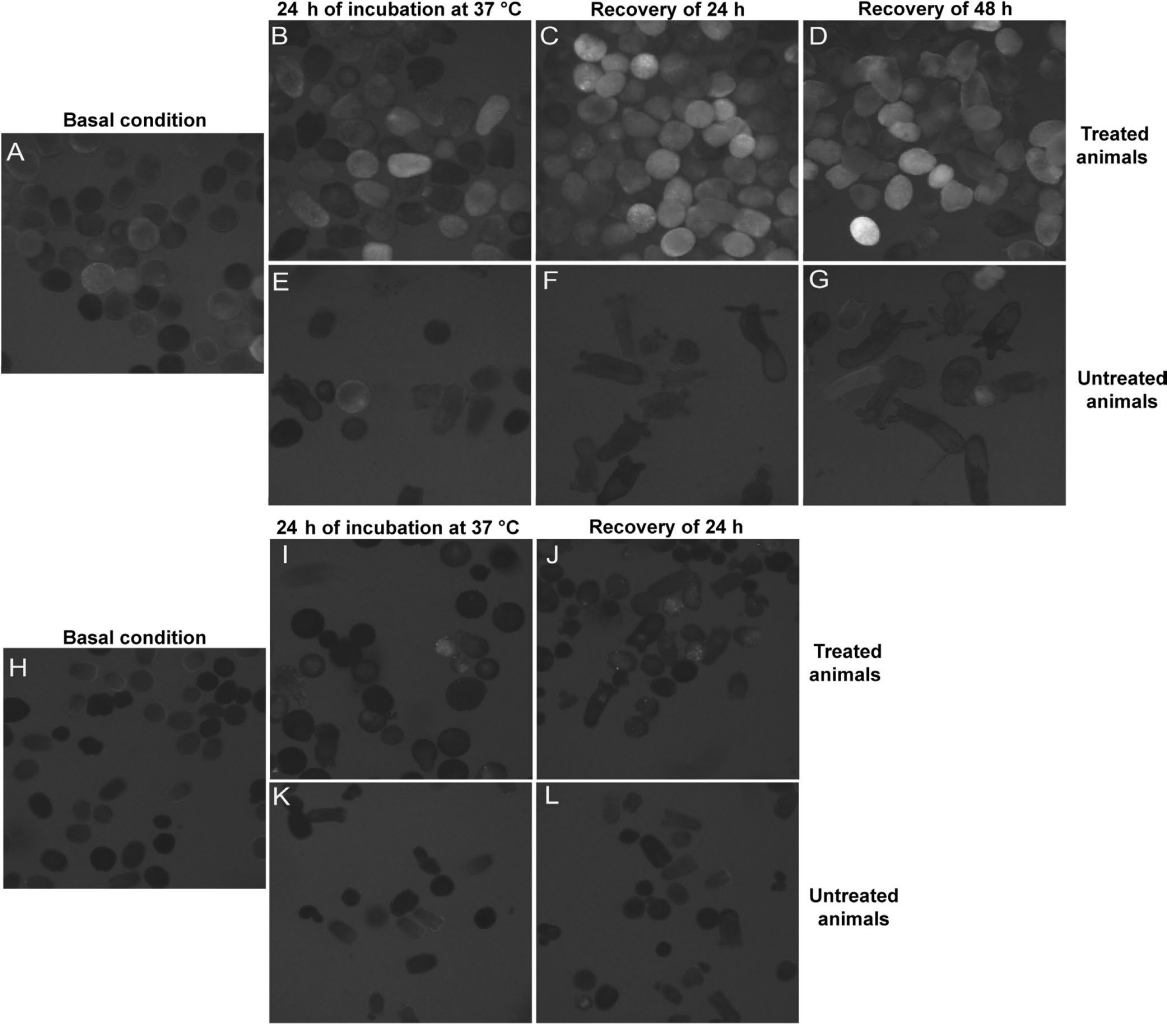
mCherry is upregulated under heat stress in the *RLRa* and *RLRb* transgenic reporter lines. 4–5 days post‐fertilisation *N. vectensis* larvae of the reporter lines were incubated for 24 h at 37°C. The larvae were given a recovery time of 19–48 h at 22°C. Untreated larvae from the same cross were used as a control group and were kept at 22°C. (A–G) panels documenting the *RLRb* line and (H–L) panels documenting the *RLRa* line. (A, H) Four‐ to five‐day‐old larvae under basal conditions (22°C) before the heat stress assay. (B, I) Five‐ to six‐day‐old larvae after 24 h of incubation at 37°C. (C, D, J) Six‐ to seven‐day‐old larvae following recovery time of 19–48 h at 22°C after the incubation of 24 h at 37°C. (E–G, K–L) Untreated animals are used as control.

We generated transgenic *N. vectensis* lines that harbour in their genome a transgenic cassette with a gene encoding the mCherry fluorescent protein (Shaner et al. 2004) downstream of the putative regulatory regions of the *RLRa* gene we predicted based on its histone marks (Figure 1A,B) (Schwaiger et al. 2014). While the transgenic cassette did not drive mCherry expression in the majority of the animals under normal conditions (Figure 1C), injection of the viral mimic poly(I:C) drove a strong fluorescent signal within 24 h post‐injection (Figure 1D,E). Similar results were obtained with a transgenic cassette containing the regulatory regions of *RLRb* (Figure 1A,F–I). The strong increase in transgene expression reflects the fact that both *RLRa* and *RLRb* are upregulated upon poly(I:C) injection (Lewandowska et al. 2021). Unexpectedly, we noticed that some of the animals injected with NaCl (a control group for the microinjection itself) also exhibited fluorescent expression, albeit at a lower level and frequency compared to the poly(I:C) group. This led us to hypothesise that stress caused by the mechanics of the microinjection and the injury it causes promotes the expression of *RLRa* and *RLRb*. To test this notion, we exposed embryos of both transgenic lines to heat stress of 37°C. Indeed, these embryos exhibited noticeable fluorescence when compared to the control group (Figure 2). Interestingly, the fluorescence was maintained for several days after the incubation.

### 3.2 Transcriptomic Analyses Highlight a Link Between Stress and Innate Immunity That Varies Across Populations

**FIGURE 3:**
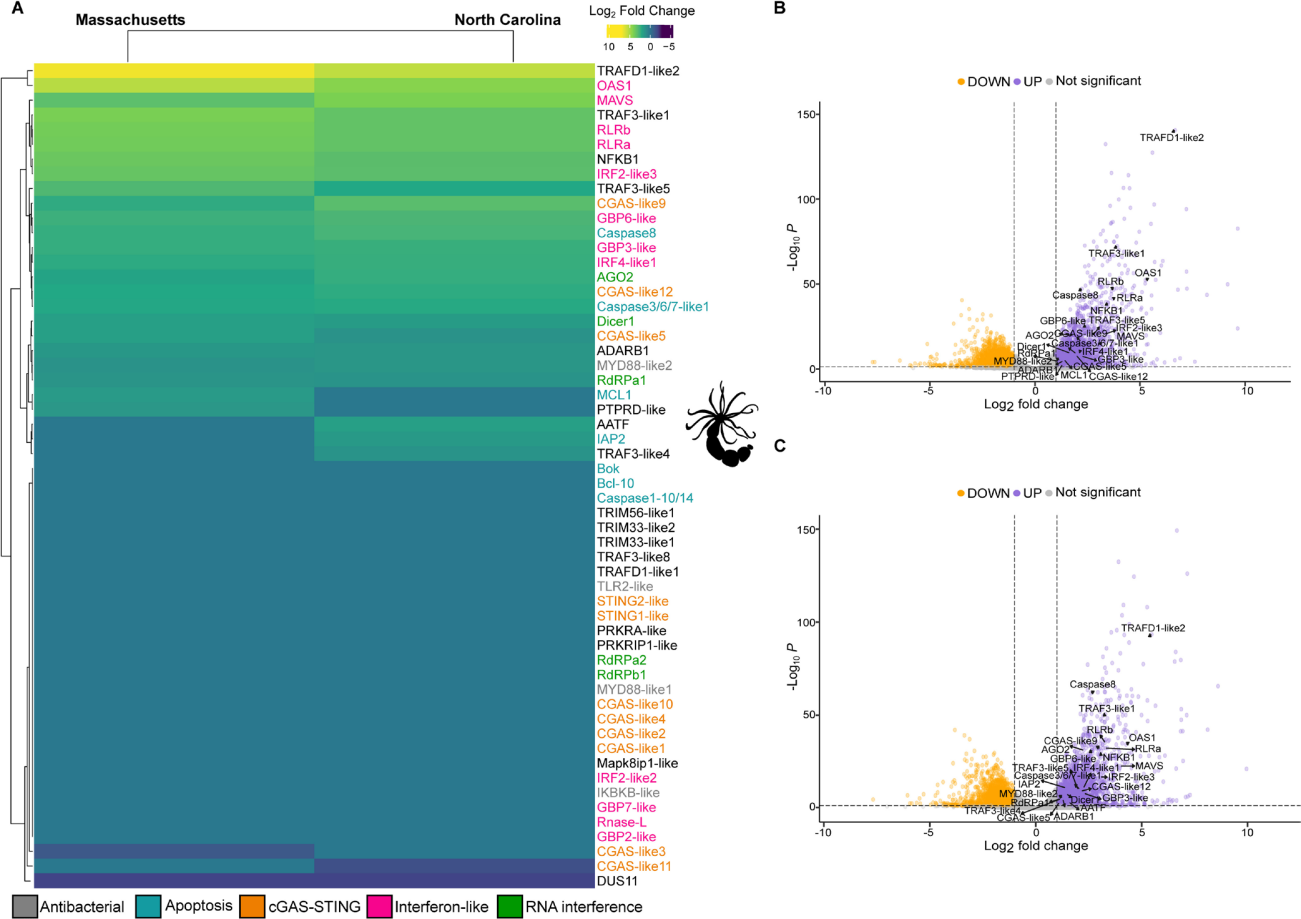
Transcriptional response of immune‐related genes in *N. vectensis* under environmental stressors, including heat stress. (A) Heatmap showing the log_2_ fold change of immune‐related genes in response to stress, comparing control and stress conditions in Massachusetts and North Carolina populations. These 56 immune genes were selected based on their known upregulation after poly(I:C) injection in *N. vectensis* zygotes (Lewandowska et al. 2021). The gene names were labelled with different colours representing the immune pathways they are involved in. Heatmap colour scale represents log_2_ fold change in gene expression. Yellow indicates strong upregulation (log_2_FC > 5), green indicates mild upregulation (log_2_FC ≈ 1) and purple indicates downregulation (log_2_FC < −5). Values range from −10 (strong downregulation) to +10 (strong upregulation). (B) and (C) Volcano plots showing differentially expressed genes (DEGs) in *N. vectensis* under combined environmental stress in the Massachusetts and North Carolina populations, respectively (Sachkova et al. 2020). Upregulated and downregulated genes (adjusted *p* value < 0.05 and absolute log_2_ fold change > 1) are highlighted in purple and orange, respectively.

**FIGURE 4:**
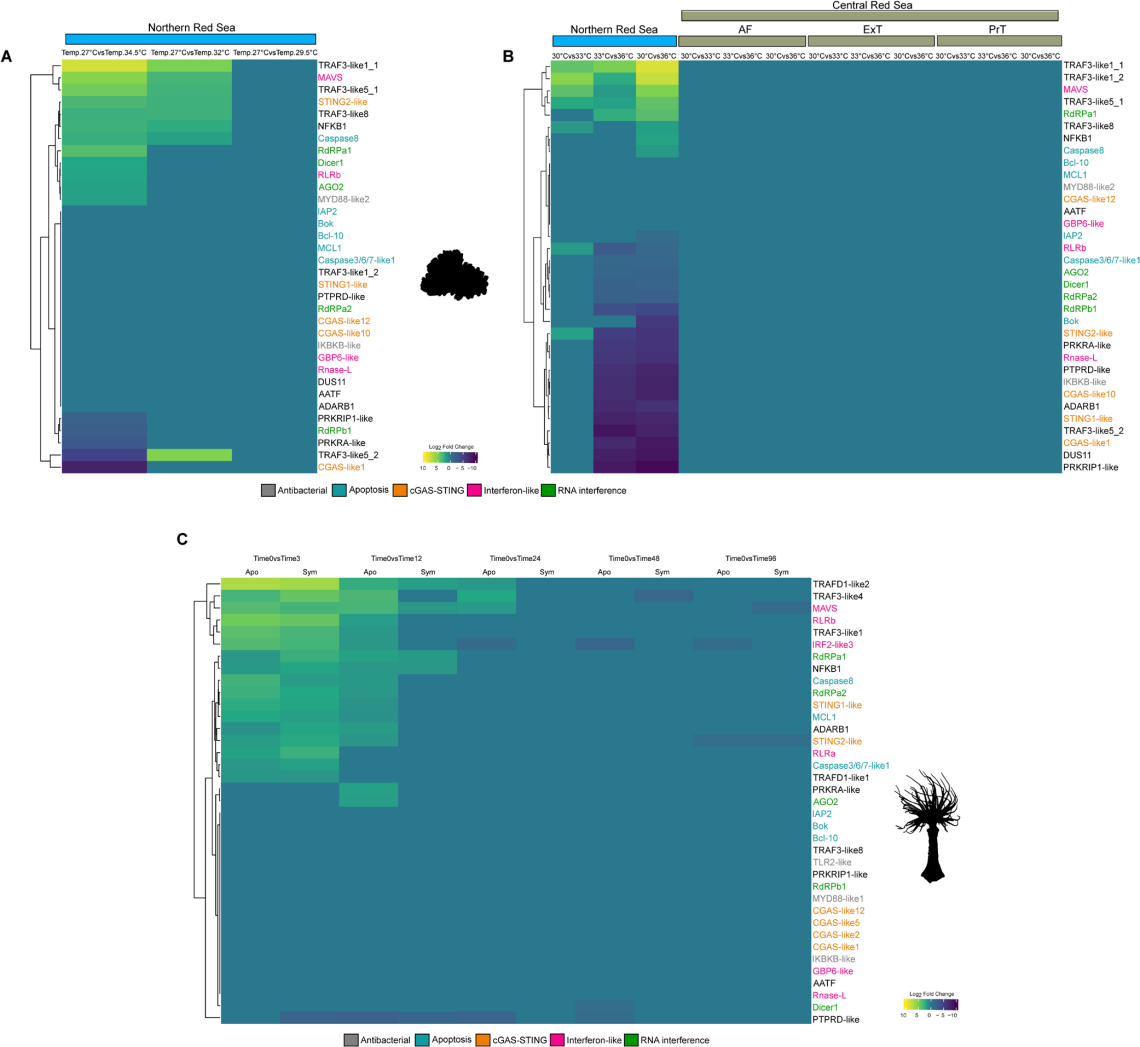
Immune response in *S. pistillata* and *E. diaphana* under heat stress. (A) and (B) Heatmaps showing the log_2_ fold change of immune‐related genes in response to heat stress in *S. pistillata* . The data are derived from two previously published studies comparing baseline and stress temperatures in populations from the Northern and Central Red Sea. The populations in the Central Red Sea include Al Fahal Reef (AF), the ocean‐facing exposed site of Tahala Reef (ExT) and the land‐facing protected site of Tahala Reef (PrT). (C) Heatmap showing the log_2_ fold change of immune‐related genes in response to heat stress, comparing baseline and stress temperatures across different time points from a previously published study on aposymbiotic (Apo) and symbiotic (Sym) of *E. diaphana* . The gene names were labelled with different colours to represent the pathways related to the immune system. Heatmap colour scale represents log_2_ fold change in gene expression. Yellow indicates strong upregulation (log_2_FC > 5), green indicates mild upregulation (log_2_FC ≈ 1) and purple indicates downregulation (log_2_FC < −5). Values range from −10 (strong downregulation) to +10 (strong upregulation).

Intrigued by the sensitivity of the *RLR* promoters to stress, we explored previously obtained transcriptomic data from *N. vectensis* adult polyps that were incubated for 24 h in harsh stress conditions which combined heat (37°C) and salinity (40‰), as well as UV light for 6 h (Sachkova et al. 2020). Indeed, more than 20 genes (part of the list of the 56 bona fide immune‐related genes) were significantly upregulated under the stress conditions in both populations (Figure 3A–C). Many of the upregulated genes were homologues of known mammalian Interferon Stimulated Genes (ISGs), including *OAS1*, *MAVS*, *RLRa*, *RLRb*, *IRF2‐like3*, *GBP6‐like*, *GBP3‐like* and *IRF4‐like1*. These genes showed log_2_ fold changes ranging from 2 to 4 (Tables S6 and S7). Notably, the anemones in this experiment were not from the common lab strain, which originates from Maryland, but from strains that originate from Massachusetts and North Carolina (Sachkova et al. 2020). Unexpectedly, despite the high similarity in the transcriptional response to stress between the two populations included in the Sachkova et al. (2020) study, transcription of several genes was altered under stress conditions in one population, but not in the other: for example, the homologue of *MCL1* (*myeloid cell leukaemia 1*), a gene known to play an important role in cell homeostasis and bypass of apoptotic reaction to stress and infection, was upregulated in the North Carolina population but not in Massachusetts. An opposite trend, upregulation in the Massachusetts strain under stress and lack of response in the North Carolina strain, was noticed for the homologue of *IAP2* (*inhibitor of apoptosis 2*), a gene that in mammals affects the apoptotic response to infections and was shown to be stress‐responsive (Dong et al. 2002). These intriguing trends demonstrate that the immune response genes of different *Nematostella* populations react differently to the same stress conditions. To test whether similar responses occur in other hexacorallians, we analysed previously obtained transcriptomic data from experiments where the sea anemone *E. diaphana* and the stony coral *S. pistillata* were exposed to heat stress conditions (Cleves et al. 2020; Savary et al. 2021). Strikingly, in both the coral (Figure 4A,B) and the sea anemone (Figure 4C), immune‐related genes exhibited significant differential expression. It was noticeable that unlike in *E. diaphana* and *N. vectensis* , in *S. pistillata* a portion of the responsive genes were downregulated under stress rather than upregulated (Figure 4A,B). More specifically, when individuals of *S. pistillata* were exposed to a stress temperature of 34.5°C, 12 genes (*MAVS, RLRb, DCR1, RdRPa1, AGO2 STING2‐like, Caspase8, TRAF3‐like8, TRAF3‐like1_1, TRAF3‐like5_1, NFKB1* and *MYD88‐like1*) were significantly upregulated (Figure 4A, Table S8), while 8 genes (*TRAF3‐like8, TRAF3‐like1_1, Caspase8, NFKB1, TRAF3‐like5_1, TRAF3‐like5_2, MAVS* and *STING2‐like*) showed upregulation in comparison to a temperature of 32°C (Figure 4A, Table S9). However, no genes were upregulated when comparing the control temperature to a stress temperature of 29°C (Figure 4A). Additionally, 5 genes (PRKRA‐like, TRAF3‐like5_2, RdRPb1, PRKRIP1‐like and CGAS‐like1) were downregulated when comparing the control temperature to 34.5°C (Figure 4A, Table S8). We chose to include the *S. pistillata* *coral* dataset due to its comprehensive experimental design, incorporating both thermal stress and geographic variation, which enabled meaningful comparisons with our findings in *N. vectensis* .

In the original experiment done on *E. diaphana* , Cleves et al. (2020) tested separately the transcriptomic response to heat stress of symbiotic (carrying zooxanthellae) and aposymbiotic (not carrying zooxanthellae) anemones. Anemones in this experiment were held at a control temperature of 27°C and a stress temperature of 34°C for different times. We identified upregulated genes only when comparing time 0 (control temperature) with 3 h and 12 h of exposure to the stress temperature in both aposymbiotic and symbiotic individuals (Figure 4C, Tables S13 and S14). Additionally, three genes remained upregulated after 24 h of exposure in the aposymbiotic individuals, that is, the response of these genes in the symbiotic group was much shorter‐lived than in the aposymbiotic group (Figure 4C).

In another transcriptomic study (Voolstra et al. 2021) that we reanalysed, the response to heat stress was documented separately for *S. pistillata* from the GoA versus the same species from the CRS. Strikingly, for the 32 bona fide immune‐response genes, in the CRS population we could find no significant differential expression, whereas in the GoA population we could find most of the genes being significantly upregulated or downregulated (Figure 4B, Tables S10–S12), similarly to the trends reported for the study by Savary et al. (2021) that also focused on the GoA population (Figure 4A).

### 3.3 Characterising Viral and Antiviral Transcription Dynamics

In order to test whether the transcriptional immune response under stress is driven, at least partially, by an increase in viral load, we analysed the RNA‐Seq data previously used in this study for *N. vectensis* (Sachkova et al. 2020) and *E. diaphana* (Cleves et al. 2020). For *N. vectensis* , we assembled viromes from Massachusetts and North Carolina populations (Smith et al. 2023) and mapped reads to these assemblies. For *E. diaphana* , reads were mapped to its published core virome (Bruwer and Voolstra 2018). While some differences in viral load between control and stress conditions were observed, they were not statistically significant, likely due to low viral read coverage resulting from polyA‐based library preparation and the high abundance of host transcripts. For these reasons, we opted not to include these results in the main study but briefly comment on them here.

**FIGURE 5:**
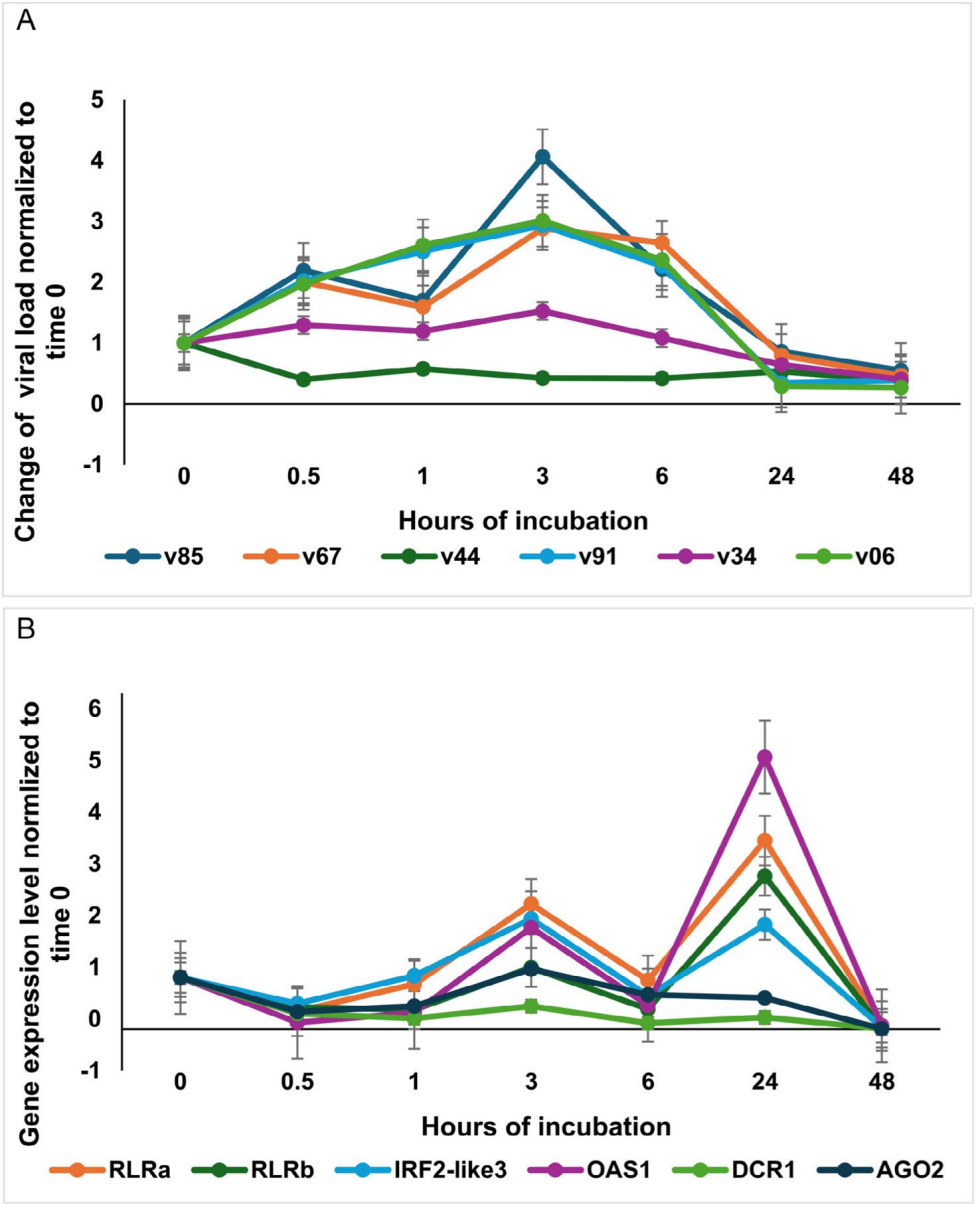
Dynamic heat stress assay for testing the antiviral immune response compared to the change of the viral load in *N. vectensis* . Adult polyps were incubated at 37°C and assayed at different time points for the expression levels of six antiviral genes (B) (Lewandowska et al. 2021) compared to six sequences from the *N. vectensis* core virome (A) (Lewandowska et al. 2020). The changes were measured by qPCR (see methods).

To circumvent these potential limitations, we decided to use a targeted approach where we amplified by specific primers six viral sequences representing the most dominant members of the core virome of *N. vectensis* and six major antiviral genes (*RLRa*, *RLRb*, *IRF2‐like3*, *OAS*, *DCR1* and *AGO2*) by quantitative PCR (qPCR; Table S2). This approach also allowed us to assay the dynamics of the stress response. We incubated adult *N. vectensis* polyps of the common lab strain to heat stress of 37°C and with samples taken at several time points. After RNA extraction and cDNA synthesis, we used qPCR to measure viral sequences as well as host antiviral gene expression. Unexpectedly, four of the viruses showed a significant load increase within 30 min from the beginning of the incubation and five of them peaked at 3 h (Figure 5A). The antiviral genes exhibited mild, yet significant, upregulation only after 1–3 h, and most of them peaked at 24 h after the start of the incubation under stress conditions (Figure 5B). Interestingly, variation in the dynamics of the responses was observed both between the antiviral genes and between the viral sequences (Figure 5A,B).

## 4 Discussion

In this work, we have set to explore how stress and immune response are linked in hexacorallians. In addition to demonstrating this link at the transcriptional level in two sea anemone species and one species of stony corals, we unexpectedly revealed that the symbiotic state in *E. diaphana* , as well as the geographic origin in *N. vectensis* and *S. pistillata* , affects the transcriptional response (Figures 3 and 4). We already reported transcriptional and proteomic differences in the response to stress between the northern and southern *N. vectensis* populations in the context of venom production (Sachkova et al. 2020). This suggests that these populations have transcriptionally adapted to different environmental conditions that typify their habitats, which is plausible in light of the extreme differences in the average temperatures and sunlight exposure between those habitats. For example, between March and December 2016, in 114 days the water temperature at the North Carolina habitat crossed 36°C whereas this happened in Massachusetts only in 39 days during the same period (Sachkova et al. 2020).

Another notable result of our analysis is that the set of immune‐related genes in *S. pistillata* from the CRS is unresponsive to heat stress while the same set responds in the population of the same coral species from the GoA (Figure 4A,B), in line with previous findings that CRS corals exhibit a measured response. Interestingly, CRS corals showed a consistent upregulation of certain stress genes also known as frontloading (Voolstra et al. 2021). Corals of the Red Sea are exceptionally heat‐resistant, yet bleaching events have increased in frequency in the central and southern sections (Monroe et al. 2018; Genevier et al. 2019). By comparison, the northern Red Sea remains largely ‘bleaching‐free’ and is sometimes presented as a ‘coral sanctuary’ from climate change (Fine et al. 2013; Osman et al. 2018) although in 2024 sporadic bleaching was observed in the northern Red Sea during the most intense marine heatwave ever experienced in this region. Overall, despite diverging over approximately 350 million years and being distributed across the globe, the three hexacorallian species maintained a conserved set of homologous immune‐related genes that likely contribute to their defence against viruses, either directly or indirectly. Moreover, in *N. vectensis* and *S. pistillata* , we observed higher baseline expression levels of these immune‐related genes in animals under basal conditions, likely acquired via adaptation to higher temperature environments.

Our transcriptomic analysis suggests that at least a partial explanation for this difference between the GoA and CRS populations may be their response to heat stress by modulating the immune system. This may be a crucial component in retaining zooxanthellae under harsh climatic conditions, especially as downregulation of immune response was previously linked to hexacorallian symbiosis with these algae (Mansfield and Gilmore 2019). It is also relevant for the difference we found between the transcriptomic responses to heat stress of immune‐related genes in aposymbiotic and symbiotic (Figure 4C) *E. pallida* , where the former group displays a prolonged response.

For discerning between the two alternative scenarios: (i) heat stress induces viruses that in turn induce the immune response or (ii) the immune system of hexacorallians evolved to be upregulated upon heat stress, posited that this would be advantageous against viruses that are activated by heat stress, we measured selected viruses (Figure 5A) and antiviral response (Figure 5B). As we detected an increase in the viral load prior to the host antiviral response in *N. vectensis* , it is plausible that the extremely swift response of the viruses to the heat stress contributes to promoting the host immune response that is lagging behind. This rapid viral activation and the consequential rapid antiviral innate immunity response by the host also make it unlikely that viral loading is a secondary response of enduring stress.

Altogether, our study reveals that diverse hexacorallians separated from one another by hundreds of millions of years of evolution respond to heat stress and upregulate a variety of homologues of immune‐related genes. Furthermore, *N. vectensis* , a major hexacorallian lab model, modulates its immune system in a matter of mere hours and that viruses putatively play a role in this response by responding even faster to the stress. Future work could focus on further untangling the effects of the stress on the host immune system from the response of the viruses, characterise the potential link to bleaching, and hopefully provide a deeper understanding of the local adaptation of hexacorallian populations and its ultimate underlying causes.

## Author Contributions

T.S., A.J.B. and Y.M. designed the research; T.S., A.J.B., M.L. and R.A. performed the research; T.S. and A.J.B. contributed new reagents or analytical tools, T.S., A.J.B. and M.L. analyzed data; M.F. and C.R.V. helped in interpretation of results. T.S. and Y.M. wrote the first draft of the paper; all authors participated in the editing of the paper.

## Conflicts of Interest

The authors declare no conflicts of interest.

## Supporting information


**Data S1:** mec70098‐sup‐0001‐DataS1.xlsx.

**Appendix S1:** mec70098‐sup‐0002‐Supinfo1.R.

**Appendix S2:** mec70098‐sup‐0003‐Supinfo2.fasta.

**Table S1:** mec70098‐sup‐0004‐TablesS1‐S15.pdf.

## Data Availability

All the data used for this work is available either in the main text or in the Supporting Information files. All sequence data are derived from public databases mentioned in the text.
