# Supplementary material for: Heat Stress Drives Rapid Viral and Antiviral Innate Immunity Activation in Hexacorallia
Source: Mol Ecol. 2025 Sep 7;34(20):e70098. doi: 10.1111/mec.70098 (PMC12530282; doi:10.1111/mec.70098)
Supplement: Supplementary file 4 — Table S1: mec70098‐sup‐0004‐TablesS1‐S15.pdf. [file MEC-34-e70098-s002.pdf]

MOLECULAR ECOLOGY

Supplemental Information for:

Heat stress drives rapid viral and antiviral innate immunity activation in Hexacorallia

Ton Sharoni[1], Adrian Jaimes Becerra[1], Magda Lewandowska[1], Reuven Aharoni[1], Christian R. Voolstra[2], Maoz Fine[1,3], Yehu Moran[1*]

Table S1. NveRLRa and NveRLRb promotor region primers, which are used for cloning and the generation of the reporter transgenic lines with mCherry.
Table S2. The sequence of primers used for qPCR.
Table S3. Statistical t-test for the dynamic heat stress assay. *N. vectensis*.
Table S4. List of orthologs identified for *E. diaphana* and *S. pistillata* through orthology analysis with OrthoFinder, based on homologs established in *N. vectensis*.
Table S5. Protein sequences of orthologous immune system-related genes were obtained for the three species analyzed in this study (*N. vectensis*, *E. diaphana*, and *S. pistillata*).
Table S6. Differential expression of immune-related genes in *N. vectensis* (Massachusetts population).
Table S7. Differential expression of immune-related genes in *N. vectensis* (North Carolina population).
Table S8. Differential expression of immune-related genes in *S. pistillata* (27°C vs 34.5°C).
Table S9. Differential expression of immune-related genes in *S. pistillata* (27°C vs 32°C).
Table S10: Differential expression of immune-related genes in *S. pistillata* (30°C vs 33°C).
Table S11: Differential expression of immune-related genes in *S. pistillata* (30°C vs 36°C).
Table S12. Differential expression of immune-related genes in *S. pistillata* (33°C vs 36°C).
Table S13. Differential expression immune-related genes in *E. diaphana* (Aposymbiotic) (Time 0h versus Time 3h and Time 0h versus Time 12h).
Table S14. Differential expression immune-related genes in *E. diaphana* (Symbiotic) (Time 0h versus Time 3h and Time 0h versus Time 12h).
Table S15. List of SRA accession numbers for the datasets used in this study.

File S1. Viral sequences from *N. vectensis* core virome (Lab population originating from Maryland) that were traced for testing the increase in the viral load
File S2. R code for differential expression analysis
